# Preliminary X-ray diffraction and ligand-binding analyses of the N-terminal domain of hypothetical protein Rv1421 from *Mycobacterium tuberculosis* H37Rv

**DOI:** 10.1107/S2053230X24005831

**Published:** 2024-06-27

**Authors:** Jihyun Park, Yu Jeong Cheon, Yoon Chae Jeong, Ki Seog Lee

**Affiliations:** a Department of Clinical Laboratory Science, College of Health Sciences Catholic University of Pusan Busan46252 Republic of Korea; b Next-Generation Industrial Field-Based Specialist Program for Molecular Diagnostics, Brain Busan 21 Plus Project, Graduate School Catholic University of Pusan Busan46252 Republic of Korea; c Ajou Energy Science Research Center, Ajou University, Suwon16499, Republic of Korea; Sungkyunkwan University School of Medicine, Republic of Korea

**Keywords:** *Mycobacterium tuberculosis*, Rv1421, Walker A/B-like motif, uridine diphosphate, UDP-*N*-acetylglucosamine

## Abstract

*Mycobacterium tuberculosis* can reside and persist in deep tissues; latent tuberculosis can evade immune detection and has a unique mechanism to convert it into active disease through reactivation. *M. tuberculosis* Rv1421 (MtRv1421) is a hypothetical protein that has been proposed to be involved in nucleotide binding-related metabolism in cell-growth and cell-division processes. However, due to a lack of structural information, the detailed function of MtRv1421 remains unclear. In this study, a truncated N-terminal domain (NTD) of MtRv1421, which contains a Walker A/B-like motif, was purified and crystallized using PEG 400 as a precipitant. The crystal of MtRv1421-NTD diffracted to a resolution of 1.7 Å and was considered to belong to either the *C*-centered monoclinic space group *C*2 or the *I*-centered orthorhombic space group *I*222, with unit-cell parameters *a* = 124.01, *b* = 58.55, *c* = 84.87 Å, β = 133.12° or *a* = 58.53, *b* = 84.86, *c* = 90.52 Å, respectively. The asymmetric units of the *C*2 or *I*222 crystals contained two or one monomers, respectively. In terms of the binding ability of MtRv1421-NTD to various ligands, uridine diphosphate (UDP) and UDP-*N*-acetylglucosamine significantly increased the melting temperature of MtRv1421-NTD, which indicates structural stabilization through the binding of these ligands. Altogether, the results reveal that a UDP moiety may be required for the interaction of MtRv1421-NTD as a nucleotide-binding protein with its ligand.

## Abstract 2

A crystal of the N-terminal domain of hypothetical protein Rv1421 from *Mycobacterium tuberculosis* H37Rv (MtRv1421-NTD) diffracted to 1.7 Å resolution. MtRv1421-NTD, which contains a Walker A/B-like motif, can bind uridine diphosphate (UDP) and UDP-*N*-acetylglucosamine, indicating that the presence of a UDP moiety on the ligand may be essential for its interaction.

## 1. Introduction

Tuberculosis (TB), which is characterized by an acute disease course and asymptomatic latent infection, is caused by *Mycobacterium tuberculosis* and represents a leading cause of morbidity and mortality worldwide (Parrish *et al.*, 1998 ▸; Bagcchi, 2023 ▸). Asymptomatic latent infection with *M. tuberculosis* can persist for years in an immune-competent host, and results in the reactivation of TB in about 5–10% of latent deep-tissue infections (Parrish *et al.*, 1998 ▸; Flynn & Chan, 2001 ▸; Balázsi *et al.*, 2008 ▸). *M. tuberculosis* undergoes a non­replicating period in macrophage phagocytes until it reactivates from latency due to reduced host immunity, which initiates the disease (Schnappinger *et al.*, 2003 ▸). The unique cell envelope of *M. tuberculosis* may be part of a defense mechanism that allows it to resist phagocytosis by the host (Lemassu & Daffé, 1994 ▸). However, the various adaptation mechanisms of the *M. tuberculosis* cell envelope against environmental changes in the host are still unclear. Therefore, understanding the key factors involved in these bacterial host-adaptation mechanisms may offer a basis for the development of novel therapeutics.

*M. tuberculosis* Rv1421 (MtRv1421) is a hypothetical protein that has been proposed to be involved in nucleotide binding-related metabolism in cell-growth and cell-division processes (Foulquier *et al.*, 2020 ▸; Jeong *et al.*, 2023 ▸). *Bacillus subtilis* YvcJ (BsYvcJ) and *Escherichia coli* RapZ (EcRapZ), as orthologous proteins to MtRv1421, are involved in the nucleotide-sugar metabolism that is required for peptido­glycan (PG) synthesis (Luciano *et al.*, 2009 ▸; Resch *et al.*, 2013 ▸; Gonzalez *et al.*, 2017 ▸). PG synthesis is initiated from uridine diphosphate *N*-acetylglucosamine (UDP-GlcNAc), which is synthesized in the cytosol via the hexosamine biosynthetic pathway (Typas *et al.*, 2012 ▸; Galinier *et al.*, 2023 ▸). In the first step of this pathway, fructose 6-phosphate and glutamine are converted to glucosamine 6-phosphate (GlcN6P) in a reaction catalyzed by GlcN6P synthetase (GlmS). Finally, UDP-GlcNAc is produced by the conversion of GlcN6P through this pathway, which is regulated by the intracellular concentrations of GlcN6P and UDP-GlcNAc (Foulquier *et al.*, 2020 ▸). At high intracellular concentrations of GlcN6P and UDP-GlcNAc in *B. subtilis*, GlcN6P binds to the ribozyme of the *glmS* transcript and stimulates its self-cleavage (Foulquier *et al.*, 2020 ▸; Galinier *et al.*, 2023 ▸). In *E. coli* the pathway contains the small regulatory RNA (sRNA) GlmZ, which stimulates translation of the mRNA encoding GlcN6P synthase. When the sRNA GlmZ binds to EcRapZ, it is inactivated through cleavage by the endoribonuclease RNaseE, thereby contributing to the regulation of glucosamine phosphate (Gonzalez *et al.*, 2017 ▸). Interestingly, the N-terminal domains (NTDs) of BsYvcJ and EcRapZ contain a Walker A/B motif with a phosphate-binding loop that participates in the binding of nucleotides (Saraste *et al.*, 1990 ▸). The Walker A motif consists of the pattern G*X*_4_GK(TS), in which the lysine residue is involved in nucleotide binding. The Walker B motif is located downstream of the Walker A motif and has a D*XX*G sequence (Luciano *et al.*, 2009 ▸). Previous studies have reported that the Walker A/B motif of BsYvcJ induces conformational changes by binding to ATP, which protects proteins from cleavage by endoproteases (Luciano *et al.*, 2009 ▸).

*M. tuberculosis*, like *B. subtilis*, possesses two conserved consecutive genes, Rv1421 and Rv1422 [which correspond to YvcJ and GlmR (formerly YvcK), respectively, in *B. subtilis*] (Foulquier *et al.*, 2020 ▸; Jeong & Lee, 2020 ▸; Jeong *et al.*, 2023 ▸), that are involved in regulating the activity of GlmS, which catalyzes the production of UDP-GlcNAc as a cytoplasmic precursor of peptidoglycan (Sperber & Herman, 2017 ▸; Foulquier *et al.*, 2020 ▸, Liang *et al.*, 2021 ▸). However, compared with BsYvcJ, the sequence identity of MtRv1421 is approximately 42%. This indicates that there is a need for further investigations into the biochemical functions of MtRv1421 in order to improve the understanding of the host-adaptation mechanism of *M. tuberculosis* at the molecular level. As a first step towards elucidating the structure of MtRv1421, it was truncated to the NTD containing the nucleotide-binding motif. In the present study, we report a high-resolution X-ray diffraction data set obtained from an MtRv1421-NTD crystal and its ligand-binding ability as determined by protein thermal shift analysis.

## 2. Materials and methods

### 2.1. Expression and purification of the protein

The NTD, consisting of residues 1–174, of MtRv1421 (with a total of 301 residues) was designed based on the NTD (residues 1–152) of the orthologous protein EcRapZ (Gonzalez *et al.*, 2017 ▸). To amplify the gene encoding Rv1421-NTD from *M. tuberculosis* H37Rv genomic DNA, the polymerase chain reaction (PCR) was performed with the forward and reverse primers containing restriction-endonuclease sites for insertion into the vector: the NdeI site in the forward primer and the HindIII site in the reverse primer are underlined in Table 1 ▸. The DNA fragments digested by NdeI and HindIII were inserted into the bacterial expression vector pET-28a(+) (Novagen, USA) to generate the plasmid pMtRv1421-NTD, which contains a 6×His tag at the N-terminus. Transformed *E. coli* Rosetta (DE3) cells containing pMtRv1421-NTD were grown in lysogeny broth (LB) medium with suitable antibiotics (50 µg ml^−1^ kanamycin and 38 µg ml^−1^ chloramphenicol) at 25°C until an optical density of 0.6 at 600 nm was attained. Overexpression of recombinant MtRv1421-NTD was induced by adding 0.5 m*M* isopropyl β-d-1-thiogalactopyranoside, followed by further growth for 18 h at 18°C. The cultured cells were centrifugated at 5000*g* for 20 min at 25°C for harvesting. Next, the harvested cell pellets were suspended in buffer *A* [50 m*M* Tris–HCl pH 8.0, 500 m*M* NaCl, 10% (*v*/*v*) glycerol] containing 1 m*M* phenylmethylsulfonyl fluoride before being disrupted by sonication at 4°C. The crude lysate was centrifuged at 25 000*g* for 20 min at 4°C. The supernatant was loaded onto a HisTrap HP column (Cytiva, USA) equilibrated in buffer *A*. The bound protein was eluted with a linear gradient of imidazole using buffer *B* [50 m*M* Tris–HCl pH 8.0, 500 m*M* NaCl, 10%(*v*/*v*) glycerol, 500 m*M* imidazole]. Fractions containing MtRv1421-NTD were purified to the final state by size-exclusion chromatography (SEC) using a HiPrep 16/60 Sephacryl S-200 HR column (Cytiva, USA) equilibrated with buffer consisting of 50 m*M* Tris–HCl pH 8.0, 150 m*M* NaCl, 10%(*v*/*v*) glycerol. The purified soluble fractions containing MtRv1421-NTD were pooled and concentrated to 11 mg ml^−1^ using an Amicon Ultra-15 centrifugal filter device (Millipore, USA). The final purified MtRv1421-NTD protein was confirmed have a purity of >95% on 15% SDS–PAGE.

### 2.2. Protein crystallization

Initial crystallization screening of MtRv1421-NTD was performed by the sitting-drop vapor-diffusion method in 96-well plates utilizing a variety of commercial screening kits such as Crystal Screen, Crystal Screen 2, PEGRx 1, PEGRx 2, Natrix, Natrix 2, SaltRx, SaltRx 2 and Index (Hampton Research, USA) at 21°C. Each 2 µl drop, consisting of protein solution and reservoir solution in equal proportions, was equilibrated against 100 µl reservoir solution. Crystals were obtained from condition 6 of PEGRx 2 (Hampton Research, USA): 0.1 *M* sodium citrate pH 5.0, 10% 2-propanol, 26%(*w*/*v*) polyethylene glycol (PEG) 400. To optimize the crystal-growth condition, drops consisting of 1.5 µl protein solution and 1.5 µl reservoir solution [0.1 *M* sodium citrate pH 5.0, 6%(*v*/*v*) 2-propanol, 16%(*w*/*v*) PEG 400] were equilibrated against 300 µl reservoir solution by the hanging-drop vapor-diffusion method using a 24-well VDXm plate (Hampton Research, USA). Table 2 ▸ provides detailed information on the crystallization conditions that gave the best-quality crystals.

### 2.3. Data collection and processing

To ensure consistent data collection under cryogenic conditions, the MtRv1421-NTD crystal was briefly immersed in a cryoprotectant solution consisting of 25%(*v*/*v*) ethylene glycol added to the reservoir solution. After flash-cooling to −180°C in a nitrogen-gas steam, diffraction data sets were collected from the MtRv1421-NTD crystal on beamline 7A at the Pohang Light Source (PLS), Pohang, Republic of Korea using an ADSC Quantum 270r CCD detector. The best crystal of MtRv1421-NTD diffracted to be a resolution of 1.70 Å. The diffraction data set from the MtRv1421-NTD crystal was processed via autoindexing to identify the unit cell and space group of the crystal and was scaled after integration of the indexed data using the *HKL*-2000 package (Otwinowski & Minor, 1997 ▸). Table 3 ▸ summarizes the details of data collection.

### 2.4. Thermal shift assay for ligand binding

A fluorescence-based thermal shift assay was performed using an Applied Biosystems 7500 Real-Time PCR System. The reactions were prepared by distributing samples into a 0.1 ml qPCR 8-strip (Gunster, Taiwan) that was sealed with cap strips. MtRv1421-NTD protein (0.15 mg ml^−1^) was equilibrated in the presence or absence of 5 m*M* of each ligand [adenosine diphosphate (ADP), adenosine triphosphate (ATP), UDP, UDP-glucose (UDP-Glc), UDP-GlcNAc, glucose-6-phosphate (G-6-P), glucosamine (GlcN), GlcNAc and GlcN-6-phosphate (GlcN6P)] in 20 µl of a reaction mixture consisting of 0.25 *M* sodium phosphate pH 7.0 (Protein Thermal Shift buffer) and 2 µl of the fluorescent SYPRO Orange (Sigma–Aldrich, USA; 5000×, diluted to 100× in water). Each melting curve was programmed with the following temperature gradient: 25°C for 2 min followed by a 1°C increase per minute from 25 to 85°C and finally maintaining the temperature at 85°C for 2 min. Significant background fluorescence was not observed in the absence of protein. The fluorescence intensity (excitation and emission at 470 and 570 nm, respectively) of SYPRO Orange was analyzed to give the melting temperature (*T*_m_) using *Protein Thermal Shift Software* version 1.6 (Thermo Fisher Scientific, USA).

## 3. Results and discussion

**Figure 1:**
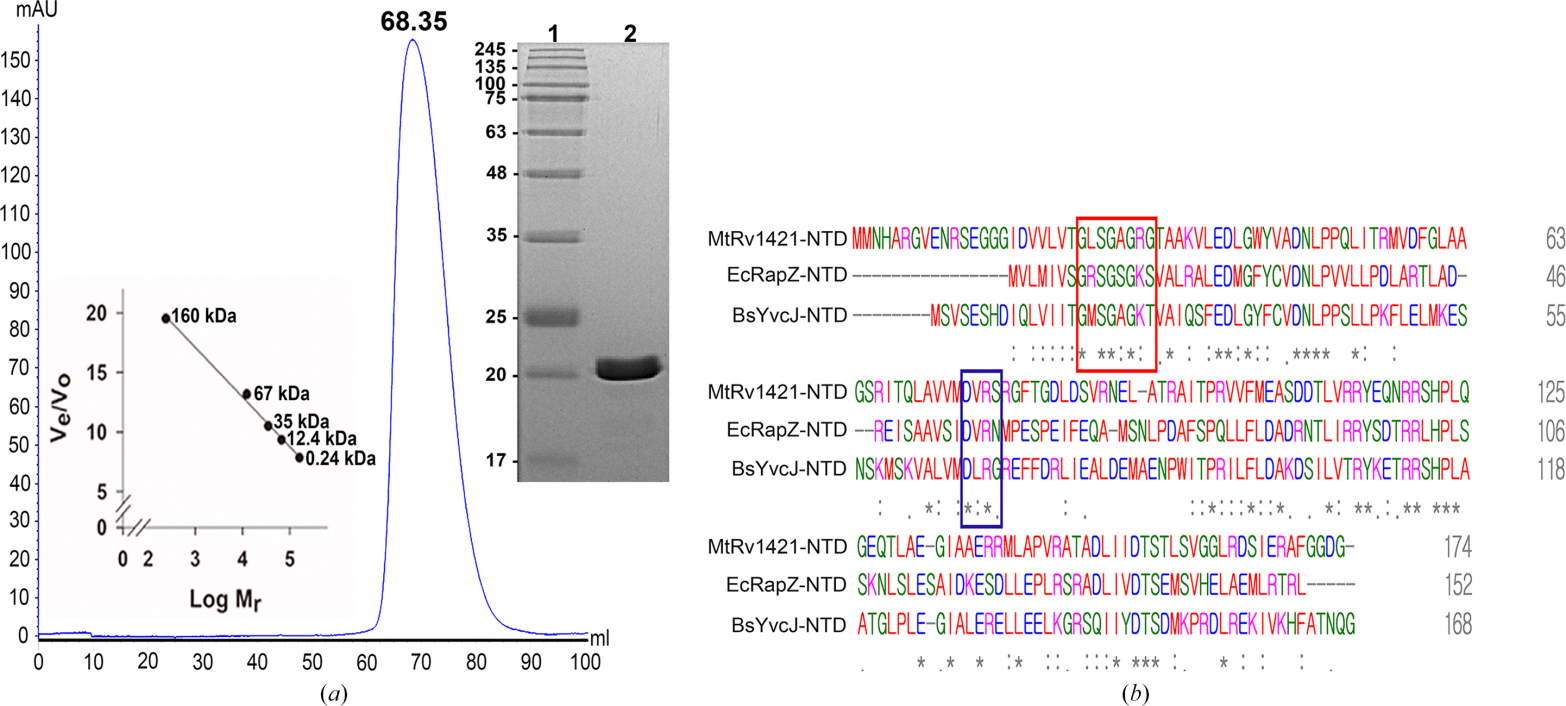
Purification of Rv1421-NTD and comparison with orthologous proteins. (*a*) SEC and 15% SDS–PAGE analyses of purified MtRv1421-NTD. The elution peak was observed at an elution volume of 68.35 ml and was expected to correspond to a monomer. 15% SDS–PAGE stained using Coomassie Blue stain; lane 1, molecular-weight markers (labeled in kDa); lane 2, purified MtRv1421-NTD (20.8 kDa). (*b*) Multiple sequence alignment of MtRv1421-NTD with EcRapZ-NTD and BsYvcJ-NTD as orthologous P-loop-containing proteins. The residues involved in the Walker A and B motifs are marked with red and blue boxes, respectively. The colored labels serve to distinguish the various residue types: red, hydrophobic; green, polar; blue, acidic; magenta, basic. The symbols used in the protein sequence alignments are asterisks for identical residues, colons for conserved residues and dots for semi-conserved residues.

The gene encoding Rv1421-NTD (residues 1–174) of *M. tuberculosis* H37Rv was successfully cloned into the pET-28a(+) expression vector and then transformed into *E. coli* Rosetta (DE3) cells. Recombinant MtRv1421-NTD was purified in a two-step process using Ni^2+^-chelated affinity chromatography and SEC. The purified MtRv1421-NTD protein showed a single band on 15% SDS–PAGE, indicating a molecular weight of 20.8 kDa, which corresponds to the calculated molecular weight for 194 residues including the His_6_ tag and vector-derived sequences (20 residues as shown in Table 1 ▸). The purity of the MtRv1421-NTD protein was estimated to be 95% or greater. The analysis using SEC showed a chromatogram peak for MtRv1421-NTD at an elution volume of 68.35 ml; therefore, it was predicted to be in the monomeric state (Fig. 1 ▸*a*). Multiple sequence alignment analysis of MtRv1421-NTD with the orthologous proteins BsYvcJ and EcRapZ containing a Walker A [G*X*_4_GK(TS)]/B (D*XX*G) motif showed that the lysine of the Walker A motif and the glycine of the Walker B motif were replaced by Arg30 and Ser78, respectively, in MtRv1421-NTD (Fig. 1 ▸*b*). This finding suggests that MtRv1421-NTD has a Walker A (G*X*_4_GRG)/B (D*XX*S)-like motif that may be involved in the binding of nucleotides, similar to the Walker A/B motif.

**Figure 2:**
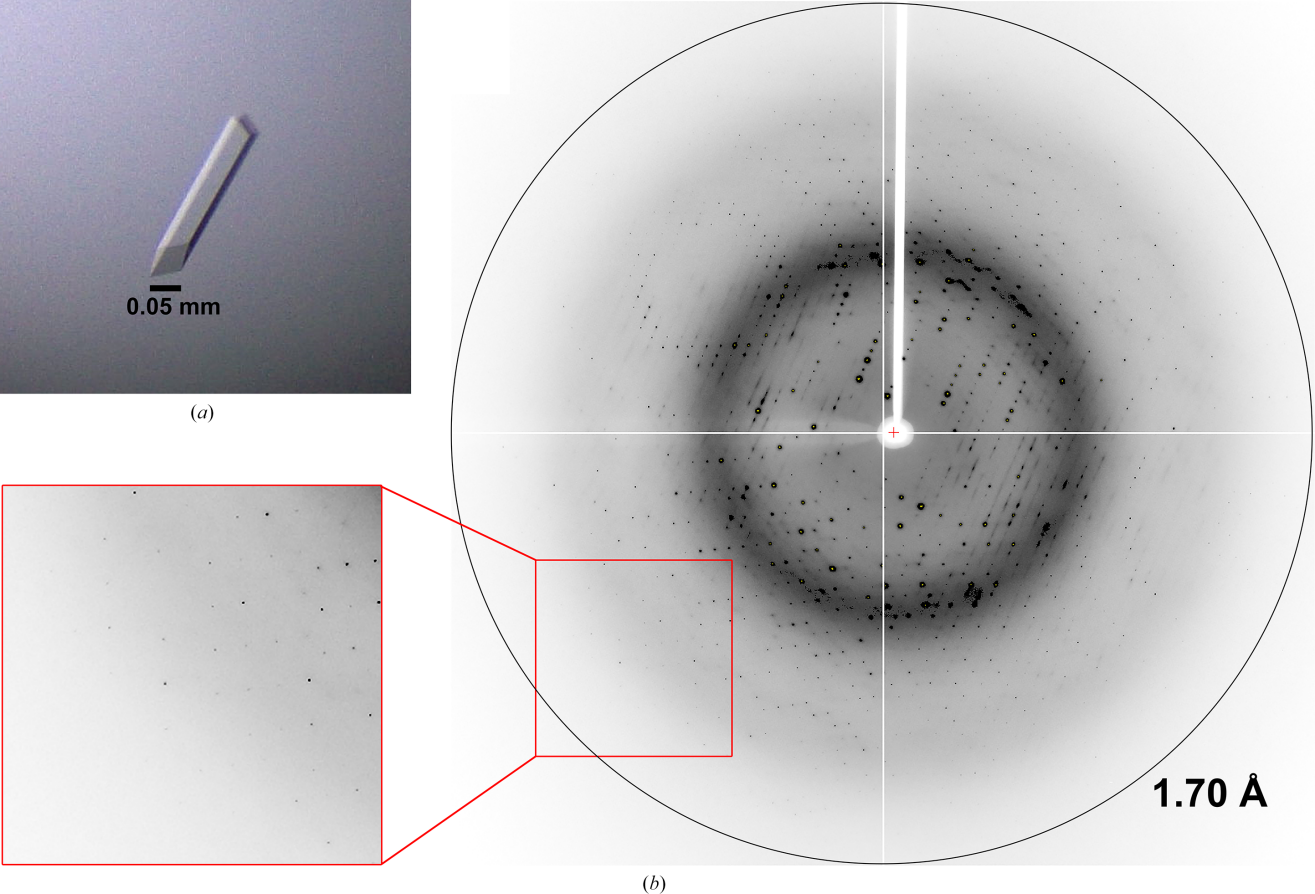
Crystal and X-ray diffraction patterns of MtRv1421-NTD. (*a*) The crystal dimensions of MtRv1421-NTD are approximately 0.05 × 0.05 × 0.3 mm for the thick rod-shaped crystal. (*b*) X-ray diffraction image of an MtRv1421-NTD crystal. The red box shows an enlarged view of the area containing high-resolution spots, where a resolution shell can be seen at 1.7 Å.

Crystals of MtRv1421-NTD were obtained within 7–10 days by the hanging-drop vapor-diffusion method using optimized reservoir solution consisting of 0.1 *M* sodium citrate pH 5.0, 6%(*v*/*v*) 2-propanol, 16%(*w*/*v*) PEG 400. The dimensions of a thick rod-shaped MtRv1421-NTD crystal were approximately 0.05 × 0.05 × 0.3 mm (Fig. 2 ▸*a*). A diffraction data set was collected from an MtRv1421-NTD crystal to a resolution limit of 1.70 Å (Fig. 2 ▸*b*). The results of the autoindexing process of X-ray diffraction images suggested two types of space group: *C*-centered monoclinic or *I*-centered orthorhombic. The former is the monoclinic space group *C*2, with unit-cell parameters *a* = 124.01, *b* = 58.55, *c* = 84.87 Å, α = γ = 90, β = 133.12°, containing two monomers in the asymmetric unit, which correspond to a Matthews coefficient *V*_M_ and solvent content of 2.7 Å^3^ Da^−1^ and 54.41%, respectively (Kantardjieff & Rupp, 2003 ▸). The latter is the ortho­rhombic space group *I*222, with unit-cell parameters *a* = 58.53, *b* = 84.86, *c* = 90.52 Å, α = β = γ = 90°, containing one monomer in the asymmetric unit, which corresponds to a Matthews coefficient *V*_M_ and solvent content of 2.7 Å^3^ Da^−1^ and 54.38%, respectively (Kantardjieff & Rupp, 2003 ▸). The value of the β angle of the unit cell is an important factor in determining the possibility of twinning or phase transition to the orthorhombic space group, and *I*-centered with the β angle close to 90° has been reported to be preferable over *C*-centered (Mighell, 2002 ▸). However, *I*222 yielded a higher value for *R*_merge_ than *C*2 in the scaling procedure (Table 3 ▸). Despite this background, we tried to find phasing solutions in both *C*2 and *I*222 by molecular replacement with *Phaser-MR* in *Phenix* (Echols *et al.*, 2012 ▸) using the crystal structure of EcRapZ (PDB entry 5o5q; Gonzalez *et al.*, 2017 ▸) as a search model. Unfortunately, none of our attempts yielded distinct solutions in either space group. To solve the phasing problem, ongoing studies will use crystals in which methionine is replaced with selenomethionine (SeMet), after which it will be possible to attempt structural determination of MtRv1421-NTD by SAD phasing.

**Figure 3:**
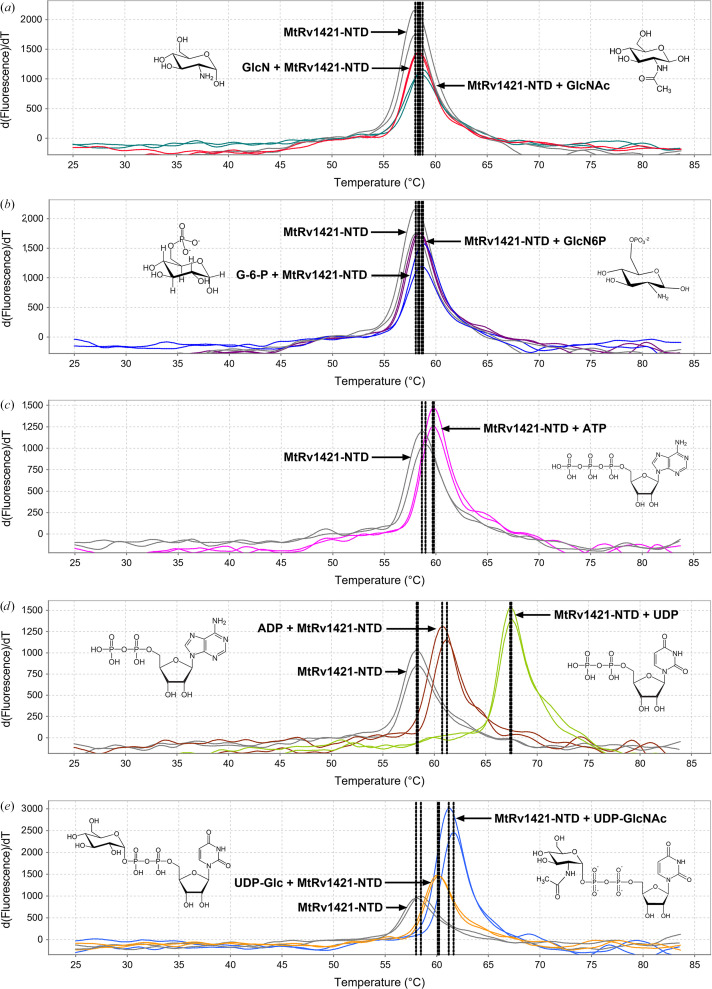
Ligand-binding ability of MtRv1421-NTD. (*a*) GlcN and GlcNAc, (*b*) G-6-P and GlcN6P, (*c*) ATP, (*d*) ADP and UDP and (*e*) UDP-Glu and UDP-GlcNAc. The plots of the difference in fluorescence versus temperature were obtained using a real-time PCR system based on four replicate reactions with and without ligands. The median derivative *T*_m_ values are indicated as black dotted vertical lines. A final concentration of 0.15 mg ml^−1^ MtRv1421-NTD was mixed with a ligand (each at 5 m*M*) along with the Protein Thermal Shift dye.

PG, which is the main component of the cell wall, consists of a three-dimensional polymer and is continuously reconstructed for the growth and survival of bacteria (Typas *et al.*, 2012 ▸). The PG precursor UDP-GlcNAc is synthesized through the action of various enzymes via the hexosamine biosynthesis pathway in the cytoplasm (Galinier *et al.*, 2023 ▸). Previous studies in *B. subtilis* have suggested that BsGlmR regulates the biosynthesis of PG precursors by interacting with BsYvcJ or GlmS, depending on the intracellular concentration of UDP-GlcNAc (Foulquier *et al.*, 2020 ▸). To verify the inter­actions of MtRv1421-NTD with nucleotides, nucleotide-sugars and amino-sugars, we conducted a ligand-binding assay using protein thermal shift. The ligand-binding ability of MtRv1421-NTD was estimated based on the shift of *T*_m_ in the presence or absence of ligand. Binding of GlcN, GlcNAc, GlcN6P and G-6-P to MtRv1421-NTD was not detected, but nucleotides and nucleotide-sugars such as ATP, ADP, UDP, UDP-Glc and UDP-GlcNAc increased the *T*_m_, indicating that the binding of these ligands contributed to structural stabilization of MtRv1421-NTD (Fig. 3 ▸). In particular, the Δ*T*_m_ value of MtRv1421-NTD for UDP was approximately 2.7-fold and 5.8-fold higher than those for UDP-GlcNAc and UDP-Glc, respectively (Table 4 ▸). These findings provide a reliable clue suggesting that MtRv1421 may be involved in the metabolic processes by which the synthesis of PG precursors is regulated. We also suggest that MtRv1421-NTD, which is a nucleotide-binding protein containing a Walker A/B-like motif, may require the presence of a UDP moiety to interact with its ligand. Further studies will involve more exhaustive trials and crystallization optimization to determine the binary structure of MtRv1421-NTD in complex with the appropriate ligands based on its ligand-binding ability.

## Figures and Tables

**Table 1:** Macromolecule-production information

Source organism	*M. tuberculosis* H37Rv
DNA source	Genomic DNA
Forward primer†	5′-GTTCATATGATGAACCATGCTAGGGGCGTC-3′
Reverse primer†	5′-TATAAGCTTCTAGCCATCACCGCCGAAGGC-3′
Cloning vector	pET-28a(+)
Expression vector	pET-28a(+)
Expression host	*E. coli* Rosetta (DE3)
NCBI Reference Sequence	NP_215937.1
Complete amino-acid sequence of the construct produced‡	MGSSHHHHHHSSGLVPRGSHMMNHARGVENRSEGGGIDVVLVTGLSGAGRGTAAKVLEDLGWYVADNLPPQLITRMVDFGLAAGSRITQLAVVMDVRSRGFTGDLDSVRNELATRAITPRVVFMEASDDTLVRRYEQNRRSHPLQGEQTLAEGIAAERRMLAPVRATADLIIDTSTLSVGGLRDSIERAFGGDG

† Restriction-enzyme sites are underlined.

‡ The extra amino acids introduced into wild-type MtRv1421-NTD by cloning are underlined.

**Table 2:** Crystallization conditions of diffracting crystals

Method	Vapor diffusion in hanging drops
Temperature (K)	294
Protein concentration (mg ml^−1^)	11.5
Buffer composition of protein solution	50 m*M* Tris–HCl pH 8.0, 150 m*M* NaCl, 10% glycerol
Composition of reservoir solution	0.1 *M* sodium citrate pH 5.0, 6%(*v*/*v*) 2-propanol, 16%(*w*/*v*) PEG 400
Volume and ratio of drop	3 µl, 1.5:1.5 ratio
Volume of reservoir (µl)	300

**Table 3:** Data-collection statistics for MtRv1421-NTD Values in parentheses are for the highest resolution shell.

Diffraction source	Beamline 7A, PLS	
Wavelength (Å)	0.979	
Temperature (K)	100	
Detector	ADSC Quantum 270r CCD	
Crystal-to-detector distance (mm)	200	
Rotation range per image (°)	1	
Total rotation range (°)	360	
Exposure time per image (s)	1	
Space group	*I*222	*C*2
*a*, *b*, *c* (Å)	58.53, 84.86, 90.52	124.01, 58.55, 84.87
α, β, γ (°)	90, 90, 90	90, 133.12, 90
Resolution range (Å)	50.0–1.70 (1.76–1.70)	50.0–1.70 (1.76–1.70)
Total No. of reflections	793400	793986
No. of unique reflections	24776	47309
Completeness (%)	97.6 (99.4)	96.4 (96.6)
Multiplicity	9.9 (6.6)	5.2 (3.5)
〈*I*/σ(*I*)〉	22.4 (5.0)	22.5 (3.6)
CC_1/2_	0.996 (0.657)	0.997 (0.473)
*R*_merge_† (%)	6.4 (42.2)	6.0 (38.9)

† *R*_merge_ = 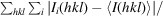

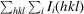
, where *I*_*i*_(*hkl*) represents the observed intensity, 〈*I*(*hkl*)〉 represents the average intensity and *i* counts through all symmetry-related reflections.

**Table 4:** Melting temperatures (*T*_m_) of MtRv1421-NTD interacting with various ligands

	*T*_m_ (°C)	Δ*T*_m_ (°C)
MtRv1421-NTD	58.36 ± 0.15	
With ligands (each at 5 m*M*)		
GlcN	58.50 ± 0.12	0.14 ± 0.12
GlcNAc	58.69 ± 0.20	0.33 ± 0.20
G-6-P	58.75 ± 0.24	0.39 ± 0.24
GlcN6P	58.67 ± 0.17	0.31 ± 0.17
ATP	59.69 ± 0.53	1.33 ± 0.53
ADP	61.31 ± 0.56	2.95 ± 0.56
UDP	67.62 ± 0.17	9.26 ± 0.17
UDP-Glc	59.96 ± 0.32	1.60 ± 0.32
UDP-GlcNAc	61.84 ± 0.31	3.48 ± 0.31
